# Cardiovascular Risk Profile of Chimeric Antigen Receptor T-cell Therapy

**DOI:** 10.7759/cureus.7436

**Published:** 2020-03-27

**Authors:** Talha Ahmed

**Affiliations:** 1 Internal Medicine, University of Maryland Medical Center, Baltimore, USA

**Keywords:** chimeric antigen receptor (car) t-cell, immuo-oncology, lymphoma, cardiac toxicity, cardiovascular effects, leukemia

## Abstract

Chimeric antigen receptor (CAR) T-cell therapy is a novel form of immunotherapy that has been recently introduced in clinical practice for the treatment of leukemias and lymphomas after being approved by United States Food and Drug Administration (USFDA). The risk profile of this treatment modality is not yet fully explored. As the survival of cancer patients is expected to rise due to new therapies being brought into practice, physicians are going to encounter side effects of these therapies. This may include both short-term and long-term effects. Having a good knowledge of these side effects can help the physicians recognize in advance the at-risk population and risk stratify them accordingly. Cardiac oncology is a growing field that involves the study of interaction between novel immunotherapies and their cardiac side effects. Knowing the cardiotoxicity profile of CAR T-cell therapy will help us choose the most appropriate patient population (those who can benefit the most without being at risk of harmful effects including cardiotoxicity) for the therapy. It will also help us recognize various cardiac complications, as they arise later during the lifetime of these cancer survivors.

## Introduction and background

Chimeric antigen receptor (CAR) T-cell therapy is a unique modality of treatment that was first approved in 2017. Initial approval led to the use of this treatment in mostly children and young adults with leukemia [1,2]. In the later years, with newer studies showing the beneficial effects of this novel therapy in adults, it was approved for the treatment of leukemias and lymphomas in adult patients as well [3]. CAR T-cell infusion protocol, however, requires close monitoring. At-risk patients particularly those with high burden of tumor cells (blast cells) or with pre-existing multiple comorbidities particularly neurological disease (like history of seizures and strokes) often require intensive care unit (ICU) monitoring. The most commonly observed side effect of CAR T-cell therapy is the cytokine release syndrome (CRS). The underlying mechanism involves massive release of cytokines in the body in response to the chimeric T-cell infusion [4]. This mainly manifests as fevers, chills, headache, nausea, vomiting and accompanying severe hypotension with tachycardia. Cardiac toxicity of CAR T cell also deserves a mention. It includes but is not limited to various arrhythmias, transient left ventricular (LV) dysfunction and troponin elevation that translates into worse outcomes like cardiogenic shock, cardiac arrest and even death from cardiac causes. Previous cardiac dysfunction and arrhythmias are not contraindications for CAR T-cell therapy. In this review, we will highlight the retrospective literature available on the cardiac toxicity of this treatment modality and also review the current prospective studies underway. 

## Review

CAR T cell is a novel form of immunotherapy which has been recently adapted in clinical practice. The term 'chimeric' synonymized as 'phantasmic' or 'unreal' very well describes this novel therapy. The patients' own T cells are first extracted via apheresis. They are genetically engineered using either lentivirus or retrovirus to express the receptor of interest (that will bind with and destroy cancerous cells in the body). CD-19 receptor, being the most prevalent receptor expressed by leukemia and lymphoma cells, is very commonly engineered into these chimeric T cells [5]. In 2017 it was approved for the treatment of children and young adults with leukemia. Subsequent studies led to the approval of the CAR T-cell therapy for adults with leukemias and certain types of non-Hodgkin lymphoma in 2018 by the United States Food and Drug Administration (USFDA).

Careful supervision is required while administering the CAR T-cell therapy. Different centers have various infusion protocols. In certain patients, especially young patients with fewer comorbidities and low cancer burden, therapy can be instituted at infusion centers that are in close proximity to major hospitals. For patients who are at high risk for developing side effects, intensive care monitoring is suitable. These include patients with a high tumor burden, those with pre-existing multiple comorbidities and with previous neurological complications [6].

Some of the well-known side effects of this treatment modality are CRS (fever, flu-like symptoms and hypotension due to massive release of cytokine), neurotoxicity (headache, seizures due to blood-brain barrier disruption), tumor lysis, various cytopenias and graft vs host disease [7]. Patients with high burden of tumor cells have high predisposition to developing CRS. This syndrome involves excessive and inappropriate release of various cytokines due to interaction between the infused chimeric T cells and hosts immune cells. This cytokine storm accounts for most of the common complications encountered while infusing CAR T cell that affect various organ systems of the body [8]. CRS is graded in severity from grade 1 to 4. Tocilizumab (a biological inhibitor of interleukin-6/IL-6 receptor) is used to treat moderate to severe CRS.

Various cardiac complications including sinus tachycardia, arrhythmias, cardiomyopathy and cardiac arrest were reported in clinical trials studying the CAR T-cell therapy. These were the pioneer studies that led to approval of this treatment. However, despite these side effects, a previous history of arrhythmias or any cardiovascular (CV) event including myocardial infarction, stroke, angina or heart failure is not an absolute contraindication to the therapy.

The first study to describe the CV effects of pediatric CAR T-cell therapy was done by Burstein et al. at the Children’s Hospital of Philadelphia in patients who received CD19-directed CAR T cells between 2012 and 2016. The primary endpoint of hypotension-requiring inotropic support occurred in 24 patients with a mean onset of 4.6 days (range, 1 to 9) after CAR T-cell infusion, including six patients receiving milrinone. Secondary endpoints included echocardiographic dysfunction at discharge and six-month follow-up that occurred in 10 patients; there were no cardiac-related deaths. The results of this study concluded that pre-treatment blast count > 25% or pre-existing cardiac dysfunction increased the risk for hypotension-requiring inotropic support [9].

Alvi et al. in their study of all 137 patients who received CAR-T between January 2016 and June 2018 measured LV ejection fraction and cardiac troponin. The outcomes of interest included CV events (defined as a combination of arrhythmia, heart failure exacerbation, cardiogenic shock and CV death). There were 17 CV events (six CV deaths, six decompensated heart failure and five arrhythmias; median time to event of 21 days). The severity of CRS correlated with worsened CV outcomes. An interesting observation made in this study was that patients with pre-existing cardiac structural abnormalities (increased left atrial size, increased LV mass or dimension) had baseline elevated troponins which translated into worse outcomes post treatment. Another reported finding of this study was that severity of CRS and delay in treating CRS with tocilizumab (1.7-fold increased risk with each 12-hour delay) led to worse CV outcomes [10]. Hence, this study postulates that elevated baseline troponin in the absence of existing acute coronary syndrome as well as severity of CRS can be considered as a surrogate for patients who are at increased risk for cardiotoxicity of CAR T-cell therapy. This advocates early management of CRS in patients who are at risk of CV events. The role of other cardiac markers, including brain natriuretic peptide and n-terminal brain natriuretic peptide, as predictors of increased risk of cardiotoxicity is not clear. 

Lefebvre et al. reviewed the charts of 90 consecutive patients undergoing CAR T-cell therapy. They observed that CV events were frequent in patients treated with CAR T cells, especially in patients with hypertension. They concluded that further prospective studies were needed to better detect and predict CV side effects and help refining treatment action and follow-up in adult patients undergoing CAR T-cell treatment [11]. 

Scherrer-Crosbie et al. (Observational [Patient Registry]: Cardiovascular Effects of CART Cell Therapy (CVE-CART); July 2021) are conducting a prospective cohort study for a period of six months that is supposed to end in 2021. Their primary outcome measures include incidence of LV, defined as a decrease in LV ejection fraction of at least 10% to less than or equal to 53% and secondary outcome measures being incidence of CV events, defined as hospitalization for symptomatic congestive heart disease, nonfatal acute coronary syndrome, CV death, nonfatal stroke and all-cause mortality. The results of this study are likely to consolidate and provide some more definitive answers in regard to the cardiac toxicity of CAR T-cell therapy observed in previous retrospective studies.

Another question of interest will be to investigate which specific group of cancer patients in particular (lymphomas vs leukemias) are more prone to the cardiac toxicity. In the study by Burstein et al., majority of the young patients (99%) had acute lymphocytic leukemia (ALL) [9]. Patients studied by Alvi et al. were mostly treated for lymphomas (88%), while the study by Lefebvre et al. also included majority of patients (90%) with lymphomas [10,11]. CAR T-cell therapy while still being investigated for acute myelogenous leukemia treatment has not yet been approved for it. Young patients with ALL after therapy mostly suffered from hypotension especially when they had a high blast cell count and pre-existing cardiac dysfunction. However, adults with lymphomas treated with CAR T cell were more likely to suffer from complications of adverse cardiac events (arrhythmias, cardiogenic shock and death from CV causes, LV dysfunction) particularly when they had a high baseline cardiac troponin and pre-existing cardiac structural and functional abnormalities. 

As the field of cardiac oncology continues to evolve with the advent of new immunotherapies, cancer survivors are expected to rise. Having a good knowledge of some of the side effects of these therapies can help the physicians recognize in advance, the at-risk population and risk stratify them accordingly. The cardiac profile of CAR T-cell therapy is not benign. Patients with multiple comorbidities with cardiac risk factors and strong cardiac history should be made aware of the potential of this therapy to affect their existing heart disease. An elevated baseline cardiac troponin should be given attention in particular. The ongoing prospective study will help answer some of the questions regarding what extent of cardiac toxicity is expected. However, since it is a six-month follow-up study, we will still not be sure about long-term effects in these cancer survivors. This will warrant a study reporting longer follow-ups to answer this question more comprehensively. 

## Conclusions

CAR T-cell therapy is an evolving therapy with promising results. Cardiac oncology is a growing field of medicine to study the cardiac risk profiles of such immunotherapies. The cardiac profile of CAR T-cell therapy is not benign. Patients with multiple comorbidities, high tumor burden and previous history of CV disease should be made aware of the potential of this therapy to affect their existing heart disease. This includes but is not limited to developing new arrhythmias and worsening of pre-existing arrhythmias as well as LV dysfunction, heart failure, cardiogenic shock requiring inotropic support and death from CV causes. Patients with pre-existing ventricular hypertrophy, increased atrial and ventricular size and elevated baseline cardiac troponin are in particular at risk of developing these complications. Moreover, the severity of CRS also correlates with worse CV outcomes in the patients who are at risk of cardiotoxicity. At the minimum, appropriate risk stratification and identification of patients who are more susceptible to these toxicities may help mitigate the cardiac toxicity of this otherwise marvelous treatment modality. 
